# Integration of Network Pharmacology and Experimental Validation to Explore the Pharmacological Mechanisms of Zhuanggu Busui Formula Against Osteoporosis

**DOI:** 10.3389/fendo.2021.841668

**Published:** 2022-01-28

**Authors:** Huihao Zhang, Chengcong Zhou, Zhiguo Zhang, Sai Yao, Yishan Bian, Fangda Fu, Huan Luo, Yan Li, Shuxin Yan, Yuying Ge, Yuying Chen, Kunyu Zhan, Ming Yue, Weibin Du, Kun Tian, Hongting Jin, Xiaofeng Li, Peijian Tong, Hongfeng Ruan, Chengliang Wu

**Affiliations:** ^1^Institute of Orthopaedics and Traumatology, The First Affiliated Hospital of Zhejiang Chinese Medical University, Hangzhou, China; ^2^Department of Pharmacy, The Second Affiliated Hospital, School of Medicine, Zhejiang University, Hangzhou, China; ^3^The Fourth Clinical Medical College, Zhejiang Chinese Medical University, Hangzhou, China; ^4^The Second Clinical Medical College, Zhejiang Chinese Medical University, Hangzhou, China; ^5^Department of Physiology, College of Basic Medical Sciences, Zhejiang Chinese Medical University, Hangzhou, China; ^6^Research Institute of Orthopedics, The Affiliated Jiang Nan Hospital of Zhejiang Chinese Medical University, Hangzhou, China; ^7^Department of Orthopedics, The First Affiliated Hospital of Zhejiang Chinese Medical University, Hangzhou, China; ^8^Department of Orthopedics and Traumatology, Shanghai Municipal Hospital of Traditional Chinese Medicine, Shanghai University of Traditional Chinese Medicine, Shanghai, China

**Keywords:** osteoporosis, Zhuanggu Busui formula, network pharmacology, pharmacological mechanisms, apoptosis, PI3K-Akt signalling

## Abstract

Osteoporosis (OP) is a common skeletal disease, characterized by decreased bone formation and increased bone resorption. As a novel Chinese medicine formula, Zhuanggu Busui formula (ZGBSF) has been proved to be an effective prescription for treating OP in clinic, however, the pharmacological mechanisms underlying the beneficial effects remain obscure. In this study, we explored the pharmacological mechanisms of ZGBSF against OP *via* network pharmacology analysis coupled with *in vivo* experimental validation. The results of the network pharmacology analysis showed that a total of 86 active ingredients and 164 targets of ZGBSF associated with OP were retrieved from the corresponding databases, forming an ingredient-target-disease network. The protein-protein interaction (PPI) network manifested that 22 core targets, including Caspase-3, BCL2L1, TP53, Akt1, *etc*, were hub targets. Moreover, functional enrichment analyses revealed that PI3K-Akt and apoptosis signalings were significantly enriched by multiple targets and served as the targets for *in vivo* experimental study validation. The results of animal experiments revealed that ZGBSF not only reversed the high expression of Caspase-3, Bax, Prap, and low expression of Bcl-2 in osteoblasts of the OP mouse model but also contributed to the phosphorylation of Akt1 and expression of PI3K, thereby promoting osteogenesis and ameliorating the progression of OP. In conclusion, this study systematically and intuitively illustrated that the possible pharmacological mechanisms of ZGBSF against OP through multiple ingredients, targets, and signalings, and especially the inhibition of the apoptosis and the activation of PI3K-Akt signaling.

## Introduction

Osteoporosis (OP), a common metabolic bone disease, is featured with decreased bone density and increased brittleness of bone, resulting in an increased risk of fracture (1, 2). It’s estimated that approximately 200 million people worldwide are suffering from OP (10.4% for males, 31.2% for females) and the number is climbing, representing a major worldwide public health problem (3, 4). The homeostasis of bone mass during bone metabolism is dynamically regulated by the osteoblast-mediated bone formation and osteoclast-mediated bone resorption (5, 6), and OP is a pathological state in which bone resorption is greater than bone formation (7, 8). Current strategies for OP fall into two categories, including anti-resorptive drugs and osteogenic drugs (9). Although these drugs are available, some are limited by side effects, such as dizziness, heart palpitations, and nausea (10). Thus, it is of importance to find new alternative therapies for OP (11).

Traditional Chinese medicine (TCM) herb formulae have their advantages and characteristics in fewer side-effects and early intervention (12, 13). “Kidney dominating bone, generating marrow” is the theoretical basis of TCM in the treatment of OP, and the deficiency of kidney function leads to OP (14). Zhuanggu Busui formula (ZGBSF) is a novel TCM formula composed of *Epimedium* (Yin Yang Huo), *Dipsaci Radix* (Xu Duan), *Paeoniae Radix Alba* (Bai Shao), *Chuanxiong Rhizoma* (Chuan Xiong), *Hedysarum Multijugum Maxim*. (Huang Qi), *Rehmanniae Radix Praeparata* (Shu Di Huang), *Eucommiae Cortex* (Du Zhong), *Achyranthis Bidentatae Radix* (Niu Xi), *Carthami Flos* (Hong Hua), *Angelicae Sinensis Radix* (Dang Gui), which is used for tonifying kidney and strengthening bone. Clinical practice has confirmed that ZGBSF is an effective prescription for treating OP. Recent system pharmacology works have shown that *Eucommiae Cortex, Dipsaci Radix and Epimedium*, several main components in ZGBSF, could promote osteogenic differentiation and ameliorate bone loss due to their anti-inflammatory and antioxidant properties (15–17). However, the underlying integrated pharmacological mechanisms of ZGBSF on OP remain largely elusive.

Because of multiple components and targets of TCM formulae, it is a challenge to explore their pharmacological mechanisms using conventional pharmacological methods (18, 19). With the rapid development of computer technology and system biology theory, network pharmacology, an emerging interdisciplinary subject, has great advantages to decipher the pharmacologic mechanisms of TCM with multi-components, multi-targets, and multi-pathways. A study reported that 16 compounds and 28 targets in Luohua Zizhu granule associated with colorectal adenoma were screened by network pharmacology, which revealed the mechanism of Luohua Zizhu granule against colorectal adenoma (20). Our previous study in the mouse model showed that apoptosis screened by network pharmacology had been validated to be the key signaling pathway of Liuwei Dihuang Decoction against intervertebral disc degeneration (21). Therefore, all evidence illustrated that network pharmacology analysis may be a good way to explore pharmacological mechanisms of ZGBSF in the treatment of OP.

**Figure 1:**
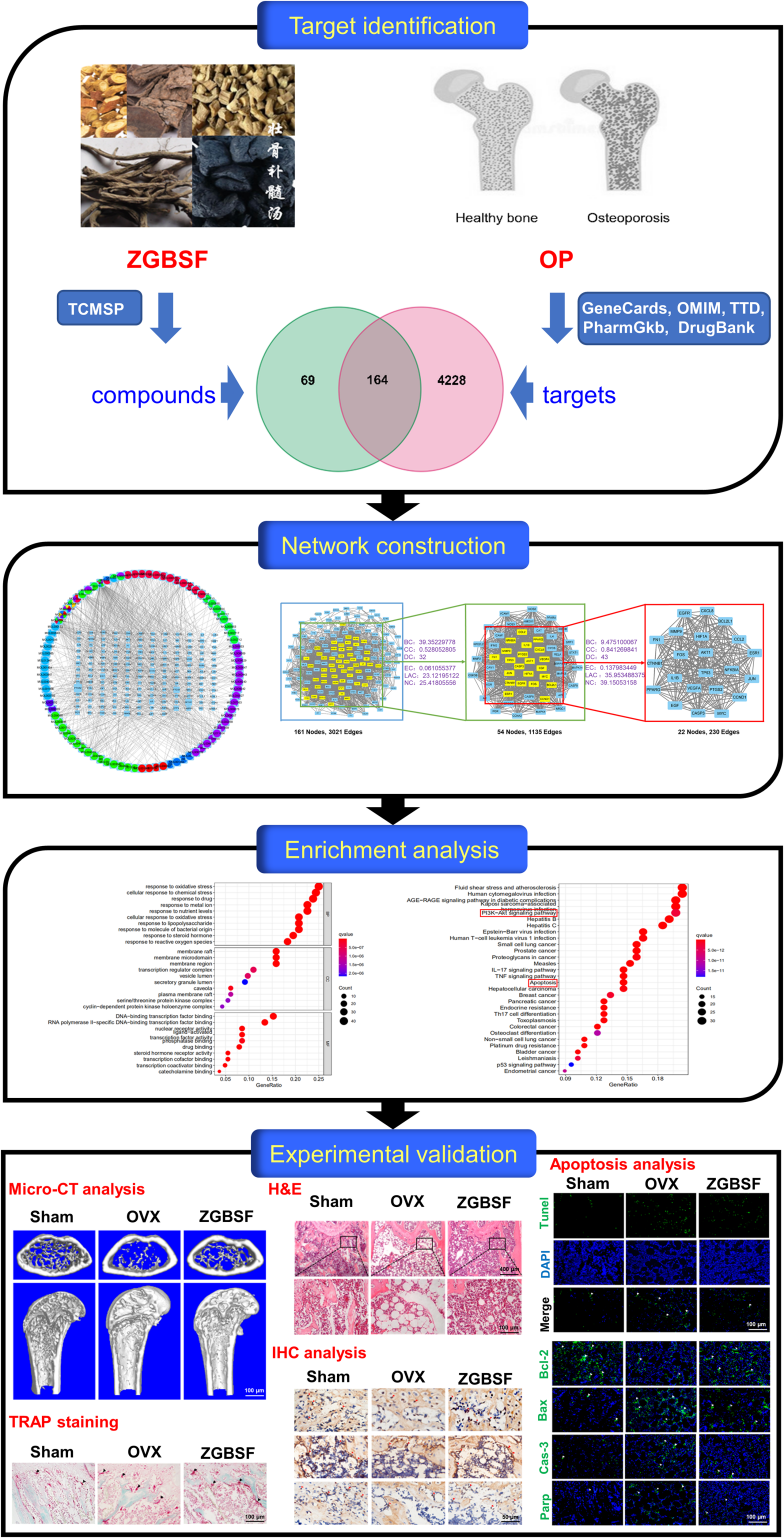
A flowchart for explaining the mechanism of ZGBSF against OP through targets identification, network construction, enrichment analysis, and experimental validation.

In this study, the active compounds of ZGBSF were firstly obtained according to the screening criteria (oral bioavailability (OB) ≥ 30% and drug-likeness (DL) ≥ 0.18) (22), and the core targets of ZGBSF associated with OP were screened by the PPI network. Next, the GO and KEGG functional enrichment analyses were utilized to screen the core signaling pathways of ZGBSF against OP. Finally, the core target proteins related to the predicted pathway were further verified in OVX mice treated with ZGBSF. A flowchart of this research was shown in **Figure 1**.

## Materials and Methods

### Screening for Active Compounds of ZGBSF and Target Prediction

The active compounds of ZGBSF were retrieved from the Traditional Chinese Medicine Systems Pharmacology (TCMSP) Database and Analysis Platform (http://tcmspw.com/tcmsp.php) (23) by using ‘Yin Yang Huo’, ‘Xu Duan’, ‘Bai Shao’, ‘Chuan Xiong’, ‘Huang Qi’, ‘Shu Di Huang’, ‘Du Zhong’, ‘Niu Xi’, ‘Hong Hua’ and ‘Dang Gui’ as keywords to screen targets correlated with ZGBSF. All target names were standardized through the UniProt database (https://sparql.uniprot.org/) (24).

### Screening of Potential Targets for OP

The potential targets related to OP were screened from DrugBank (https://go.drugbank.com/) (25), Genecards (https://www.genecards.org/) (26), Therapeutic Target Database (TTD, http://bidd.nus.edu.sg/group/ttd/ttd.asp) (27), PharmGkb (https://www.pharmgkb.org/) (28) and Online Mendelian Inheritance in Man (OMIM, https://www.genecards.org/) (29). After elimination of repetitive targets, the potential targets correlated with OP were obtained.

### Drug-Target-Disease Network Construction

A drug-target-disease interaction network was constructed by inputting active compounds in ZGBSF and common targets of these active compounds and OP into Cytoscape 3.8 software. The degree was used to evaluate the importance of compounds and targets, which represented the total number of routes associated with the node and other nodes. And the higher the degree, the more important it was (30).

### Protein-Protein Interaction Network

To clarify the functional interactions among the screened potential proteins, they were all imported to the String database (https://string-db.org/) to construct Protein-Protein Interaction (PPI) network (31). Then the PPI network was inputted into Cytoscape 3.8 software, and key proteins were identified by CytoNCA software according to betweenness centrality (BC), closeness centrality (CC), degree centrality (DC), Eigenvector centrality (EC), local average connectivity-based method (LAC), and network centrality (NC), which were greater than or equal to the median value (32).

### Functional Enrichment Analyses

The GO and KEGG functional enrichment analyses were performed through R software to obtain the core biological processes and signaling pathways. The top 10 items of GO analysis and 30 items of KEGG analysis were mapped as bubble plots. Finally, R software was utilized to generate the key signaling pathways related to OP (33).

### Chemicals and Reagents

Primary antibodies against total (t)-Akt1, Bcl-2, Bax, Parp were purchased from Ruiying Biological Co. (Jiangsu, China). Primary antibodies against Caspase-3, p-Akt1(S473), PI3K were obtained from Hua’an Biological Co. (Hangzhou, China). Primary antibodies against alkaline phosphatase (Alp), Osterix (Osx) were from CUSABIO Biological Co. (Wuhan, China). And primary antibody against Runx2 was supplied by Abcam Company Ltd. (Cambridge, MA, USA). Alexa Fluor^®^ 488 rabbit anti-goat IgG (H+L) secondary antibody (green) was obtained from Zhongshan Jinqiao Biotech Co. (Beijing, China). TUNEL Bright Green Apoptosis Detection Kit was supplied by Vazyme Biotech Co. (Nanjing, China). The remaining chemicals were from Sigma-Aldrich (St. Louis, Mo, USA) unless otherwise stated.

### Preparation of ZGBSF

Ten raw herbs of ZGBSF were provided from the pharmacy department of the first affiliated hospital of Zhejiang Chinese Medical University (Hangzhou, China). A voucher of the specimen was stored at the first affiliated hospital of Zhejiang Chinese Medical University (**Table 1**). *Epimedium, Dipsaci Radix, Paeoniae Radix Alba, Chuanxiong Rhizoma, Hedysarum Multijugum Maxim., Rehmanniae Radix Praeparata, Eucommiae Cortex, Achyranthis Bidentatae Radix, Carthami Flos, Angelicae Sinensis Radix* were mixed as a proportion of 2:1.5:1.5:1:1.5:2:1.5:1.5:1:1.5 (w/w), and then soaked in 12 volumes of distilled water (v/m) for 1 h, decocted for 2 times, and 1.5 h each time. Finally, the water-based decoction was concentrated to 1 g/mL and stored at -20°C prior to use.

**Table 1:** Detailed information of Zhuanggu Busui formula (ZGBSF).

Chinese Name	Latin Name	Parts Used	Place of Origin	Voucher Specimen NO.
Yin Yang Huo	*Epimedium*	Stem Leaf	Shanxi, China	161100
Xu Duan	*Dipsaci Radix*	Root	Jiangxi, China	161101
Bai Shao	*Paeoniae Radix Alba*	Root	Guangxi, China	161102
Chuan Xiong	*Chuanxiong Rhizoma*	Root Stem	Sichuan, China	161103
Huang Qi	*Hedysarum Multijugum Maxim*.	Root	Shanxi, China	161104
Shu Di Huang	*Rehmanniae Radix Praeparata*	Root	Henan, China	161105
Du Zhong	*Eucommiae Cortex*	Bark	Guangxi, China	161106
Niu Xi	*Achyranthis Bidentatae Radix*	Root	Anhui, China	161107
Hong Hua	*Carthami Flos*	Flower	Henan, China	161108
Dang Gui	*Angelicae Sinensis Radix*	Root	Gansu, China	161109

### Animals and Experimental Grouping

Twenty-four C57BL/6J female mice (8-week-old, 20 ± 1 g) were provided by the animal center of Zhejiang Chinese Medical University (Hangzhou, China) and housed at the Animal Care Facility of Zhejiang Chinese Medical University according to the institutional guidelines for laboratory animals. All proposals concerning animals were approved by the Ethics Committee of Zhejiang Chinese Medical University (20211220–02), and all laboratory procedures were performed following the Regulations for the Administration of Affairs Concerning Experimental Animals approved by the State Council of the People’s Republic of China.

### OVX Modeling and Treatments

All mice were randomly divided into 3 groups: Sham group, Ovariectomy (OVX) group, ZGBSF group (n = 8 in each group). After anesthesia with 1.5% isoflurane (R510-22-16, RWD Life Science, Shenzhen, China) in 100% oxygen flowing at 1.0 L/min, two ovaries of OVX and ZGBSF mice were removed, while the ovaries of Sham mice were preserved, and only part of the fat tissue around the ovaries was removed. Two days after OVX surgery, mice in the ZGBSF group were orally administered with ZGBSF (0.2 mL/10 g body weight), once a day for 8 consecutive weeks. Mice in the other two groups were given the same dosage of normal saline. All mice were euthanized 8 weeks after intragastric administration, and the femurs were collected for further analysis.

### Micro-CT Analysis

After fixation with paraformaldehyde, the femurs were scanned using micro-CT (μCT) equipment (Skyscan 1176, Bruker μCT N.V., Kontich, Belgium) at a voltage of 50 kV with a current of 500 μA and a resolution of 9 μm per pixel. The NRecon v1.6 and CTAn v1.15 software were utilized to reconstruct the three-dimensional (3D) structure of the distal femoral metaphysis as we previously described (34).

### Histological, Immunohistochemistry, and Immunofluorescence Analyses

After μCT analysis, the femurs were decalcified with 14% EDTA (pH 7.4) for 3 weeks, then dehydrated, embedded in paraffin, and processed into 5-μm-thick coronal-oriented sections. Then sections were stained with hematoxylin-eosin (H&E) and tartrate-resistant acid phosphatase (TRAP) as we previously described (35, 36). For immunohistochemistry (IHC) or immunofluorescence (IF) analysis, sections were incubated with primary antibodies of Runx2 (diluted 1:300), Alp (diluted 1:200), Osx (diluted 1:300), t-Akt1 (diluted 1:300), p-Akt1(S473) (diluted 1:50), PI3K (diluted 1:50), Bcl-2 (diluted 1:300), Bax (diluted 1:300), Caspase-3 (diluted 1:300), Parp (diluted 1:300) at 4°C overnight. Secondary biotinylated goat anti-rabbit antibody (diluted 1:1000) (Invitrogen) was added for 30 min the following day, and a diaminobenzidine solution (Invitrogen) was used to detect IHC staining. For IF analysis, secondary goat anti-rabbit antibody conjugated with fluorescence (diluted 1:1000) was added for 30 min the next day. The quantification of positive cells was estimated in a blinded manner with Image-Pro Plus 6.0 (Media Cybernetics, Silver Spring, MD, USA).

### TUNEL Assay

To further evaluate apoptosis of osteoblasts in femoral tissues, the DNA damage, another hallmark of aopotosis, was determined by the TUNEL Bright Green Apoptosis Detection Kit (Vazyme Biotech, Nanjing, China). All operations were performed according to the manufacturer’s instructions. The Image-Pro Plus 6.0 (Media Cybernetics, Silver Spring, MD, USA) was used to calculate the number of positive cells by using three sections from each sample in three randomly selected fields of view with blind method. And the total number of cells was calculated by DAPI staining.

### Statistical Analysis

All numerical data were expressed as mean ± standard error of mean (SEM). One-way analysis of variance (ANOVA) with a Student-Newman-Keuls (SNK) *post hoc* test was performed using GraphPad Prism 6.0 (GraphPad Software Inc., La Jolla, CA). When *P* < 0.05, the differences were considered to be statistically significant.

## Results

### Active Compounds in ZGBSF and Targets Prediction

A total of 145 active compounds of ZGBSF were screened, including 23 from Yin Yang Huo, 8 from Xu Duan, 13 from Bai Shao, 7 from Chuan Xiong, 20 from Huang Qi, 2 from Shu Di Huang, 28 from Du Zhong, 20 from Niu Xi, 22 from Hong Hua, and 2 from Dang Gui. And 2729 related targets of ZGBSF were obtained from the TCMSP database, containing 511 types in Yin Yang Huo, 63 types in Xu Duan, 123 types in Bai Shao, 42 types in Chuan Xiong, 462 types in Huang Qi, and 34 types in Shu Di Huang, 532 types in Du Zhong, 444 types in Niu Xi, 449 types in Hong Hua, and 69 types in Dang Gui. After excluding duplicates, a total of 86 compounds and 233 targets were selected for the following research.

### Potential Targets Prediction of OP

**Figure 2:**
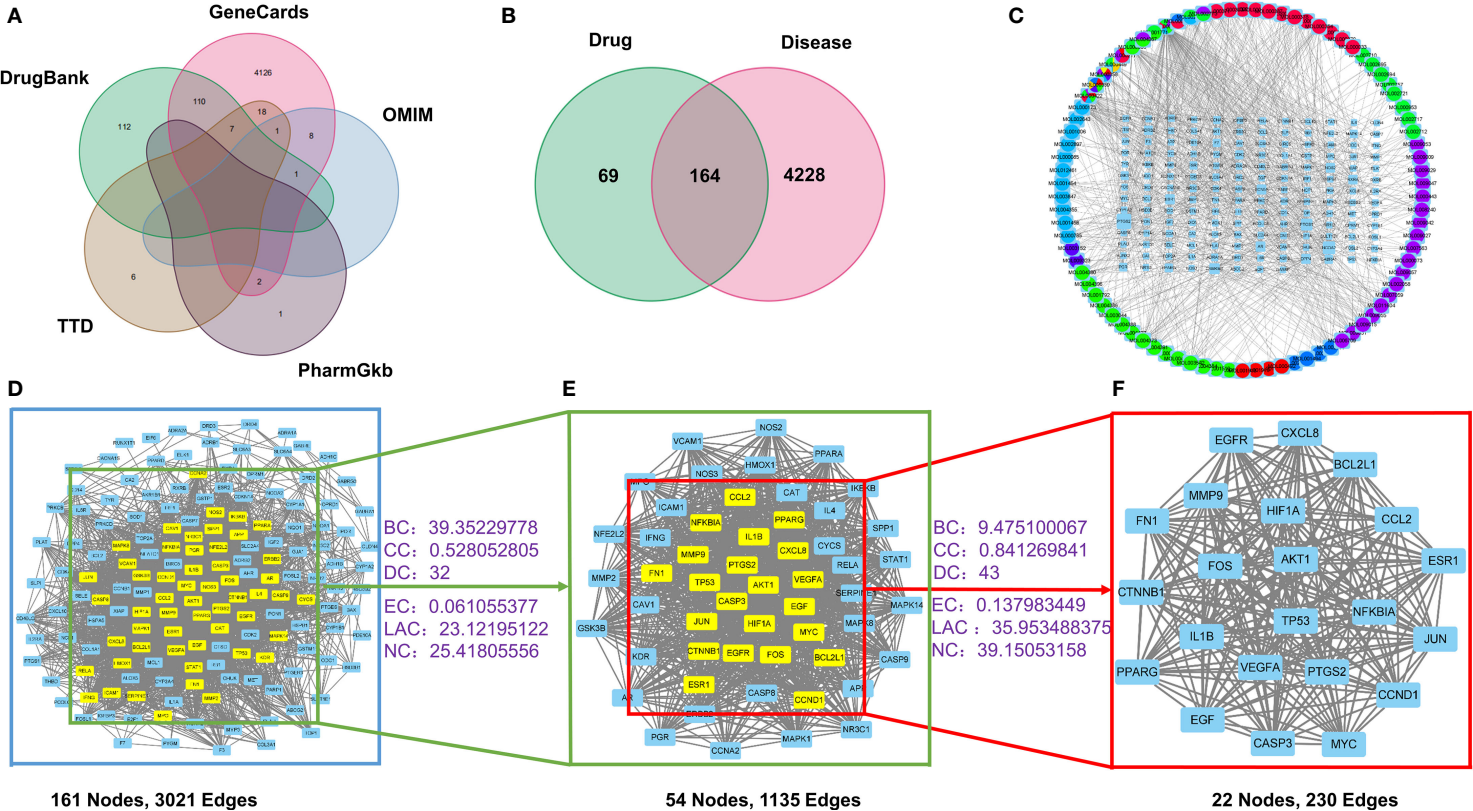
The drug-target-disease network and PPI network construction. **(A)** The collection of predictive targets for OP. **(B)** The intersection targets of ZGBSF-related targets and OP-related targets. **(C)** The network of drug-target-disease includes 10 kinds of herbs, 86 active components, and 164 target genes. Circle represents active components and rectangle represents related targets. **(D)** PPI network of predicted targets of ZGBSF against OP. **(E)** The significant proteins of the PPI network were extracted from **(D)**. **(F)** Screening 22 key proteins of ZGBSF in the treatment of OP were extracted from **(E)**.

To identify potential targets of OP, we searched 5 databases for OP-related research reports. A total of 4725 targets of OP were screen out, of which 384 were from the DrugBank database, 4273 from the GeneCards database, 31 from the TTD database, 3 from the PharmGkb database, 34 from the OMIM database. After expurgating duplicate targets, 4392 targets related to OP were screened out (**Figure 2A**).

### Drug-Target-Disease Network Construction

To obtain the intersection of ZGBSF target genes and OP target genes, the Venn diagram analysis was performed. The results showed that a total of 164 target genes were identified (**Figure 2B**), which matched with the related targets of 86 active compounds. Then, to clarify the relationship among active compounds, targets, and OP, a drug-target-disease network, including 250 nodes and 847 edges, was constructed by using Cytoscape 3.8 software (**Figure 2C**). The top 10 core compounds of ZGBSF against OP were listed in **Table 2**.

**Table 2:** The top 10 key compounds of ZGBSF against OP.

Molecular ID	Ingredient	Degree	Source	OB(%)	DL
MOL000098	quercetin	108	Du Zhong, Hong Hua, Huang Qi, Niu Xi, Yin Yang Huo	46.43	0.28
MOL000006	luteolin	45	Hong Hua, Yin Yang Huo	36.16	0.25
MOL000422	kaempferol	38	Bai Shao, Du Zhong, Hong Hua, Huang Qi, Niu Xi, Yin Yang Huo	41.88	0.24
MOL000173	wogonin	35	Niu Xi	30.68	0.23
MOL002714	baicalein	25	Hong Hua, Niu Xi	33.52	0.21
MOL000378	7-O-methylisomucronulatol	24	Huang Qi	74.69	0.30
MOL000392	formononetin	23	Huang Qi	69.67	0.21
MOL000354	isorhamnetin	22	Huang Qi	49.60	0.31
MOL004373	Anhydroicaritin	21	Yin Yang Huo	45.41	0.44
MOL004391	8-(3-methylbut-2-enyl)-2-phenyl-chromone	19	Yin Yang Huo	48.54	0.25

### PPI Network Construction and Key Targets

To obtain the pivotal proteins of ZGBSF in treating OP, after removing 3 isolates, a PPI network with 161 nodes and 3021 edges was constructed by using a String database (**Figure 2D**). Then, the thresholds of BC, CC, DC, EC, LAC, and NC were set in two steps according to the topological parameters calculated by CytoNCA. And a total of 22 pivotal proteins were filtered out, including Caspase-3, BCL2L1, TP53, IL-1β, Akt1, ESR1, FN1, CCND1, VEGFA, HIF1A, EGFR, CXCL8, CCL2, CTNNB1, JUN, PPARG, FOS, NFKBIA, PTGS2, MYC, MMP9, EGF, which were strongly linked to OP (**Figures 2E, F**).

### Functional Enrichment Analysis

**Figure 3:**
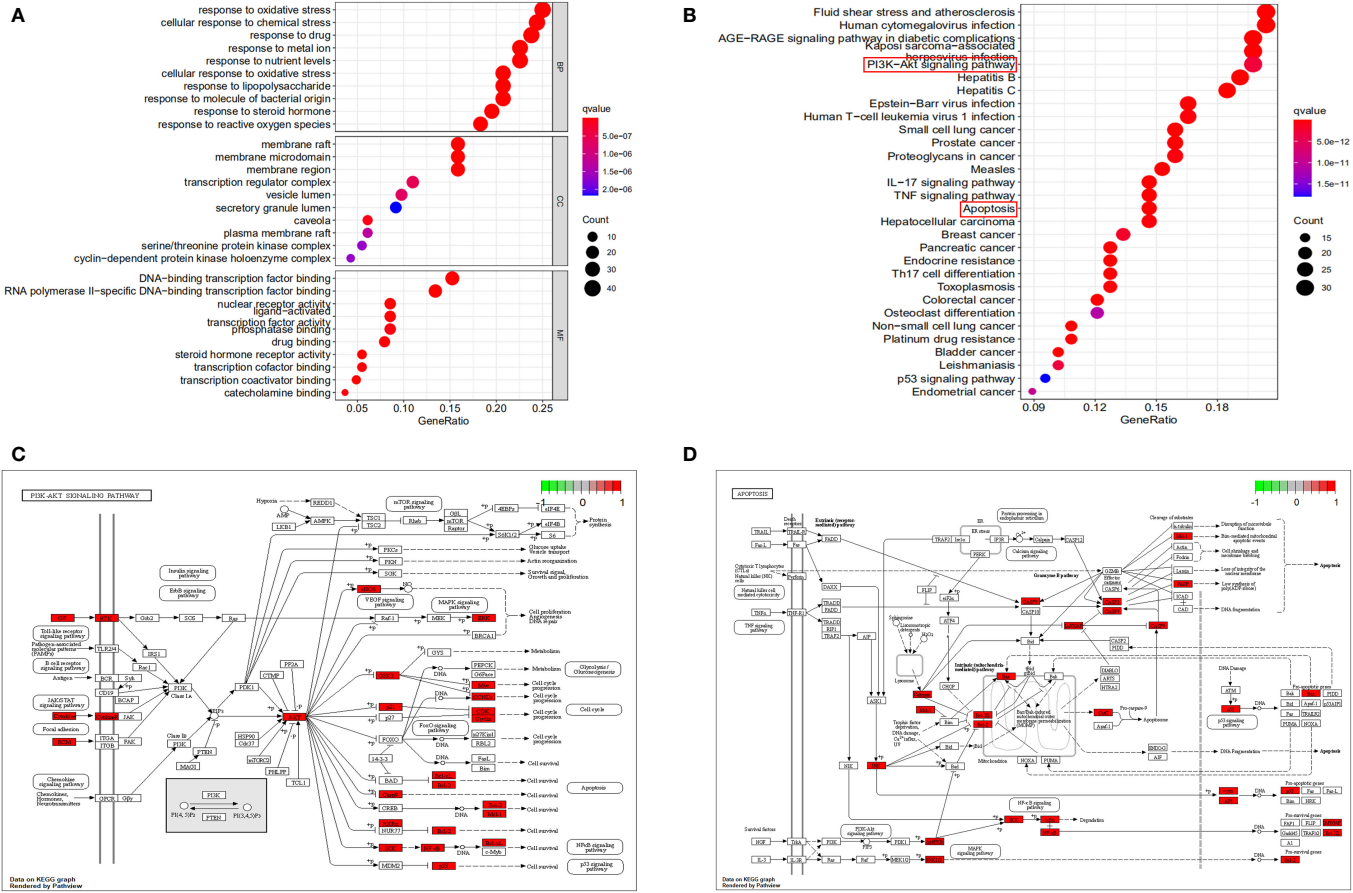
GO and KEGG functional analysis. **(A)** The top 10 of GO enrichment analysis in Biological processes (BP), cellular components (CC) and molecular function (MF). **(B)** The top 30 signaling pathways were analyzed by KEGG. **(C, D)** The PI3K-Akt signaling pathway **(C)** and apoptosis **(D)** were described in detail. The larger the nodes, the darker the color, and the more genes were enriched.

To explore the crucial biological processes of ZGBSF in the treatment of OP, the GO functional enrichment analyses were performed. The results of GO analysis revealed that the 164 genes were enriched in 2709 GO entries, consisting of 2401 biological progress (BP), 92 cellular components (CC), and 216 molecular functions (MF) (*P* < 0.05), and the top 10 entries of BP, CC and MF were shown in **Figure 3A**. BP analysis revealed that related targets were mainly centered on cellular response to chemical stress, drug, oxidative stress, *etc*. CC analysis indicated that related targets were mainly centered on membrane microdomain, membrane raft, *etc*. Moreover, MF analysis showed that potential targets were primarily focused on nuclear receptor activity, transcription factor activity, RNA polymerase II−specific DNA−binding transcription factor binding, *etc*.

To investigate the representative signaling pathways associated with the key targets, the KEGG enrichment analysis was performed. And the results showed that 160 significantly enriched signaling pathways were retrieved (*P* < 0.05). The top 30 significantly enriched signaling pathways closely correlated with OP were shown in **Figure 3B**, including apoptosis, PI3K-Akt, and TNF signaling pathways, *etc*. Furthermore, we found that apoptosis and PI3K-Akt signaling pathways were most closely related to OP in these enriched pathways, and the predicted targets corresponding to these two signaling pathways were shown in **Figures 3C, D**.

### ZGBSF Ameliorates Bone Loss and the Progression of OP in OVX Mice

**Figure 4:**
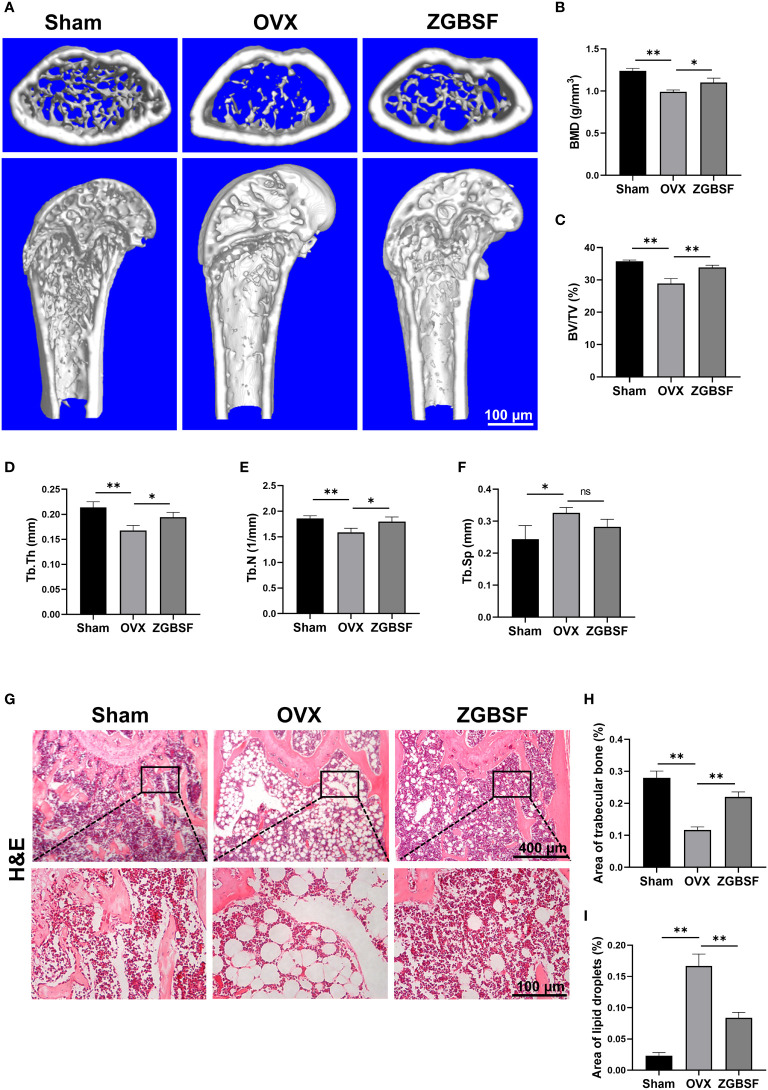
ZGBSF ameliorates bone loss in OVX mice. **(A)** Representative μCT images of distal femoral. Quantification of bone mineral density (BMD) **(B)**, bone volume fraction (BV/TV) **(C)**, trabecular thickness (Tb.Th) **(D)**, trabecular number (Tb.N) **(E)** and trabecular separation (Tb.Sp) **(F)**. **(G)** Hematoxylin-eosin staining of the distal femur. **(H)** The area of trabecular bone (%). **(I)** The area of lipid droplets (%). Data were presented as mean ± SEM of three independent experiments. *P < 0.05, **P < 0.01, NS indicated no significant difference.

To verify the core pharmacological mechanism of ZGBSF in treating OP predicted by the above-mentioned network pharmacology analysis, OVX model mice were established and treated intragastrically with ZGBSF for 8 weeks. The femurs of OVX mice were harvested and radiographic alterations were assessed by μCT analysis. The results of μCT analysis showed that compared with sham mice, the bone mass of OVX mice significantly reduced, whereas ZGBSF intervention significantly restored the bone mass (**Figure 4A**). Moreover, the results of bone morphological parameter analysis showed that ZGBSF enhanced the decrease of bone mineral density (BMD), bone volume fraction (BV/TV), trabecular thickness (Tb.Th), trabecular number (Tb.N) in OVX mice. However, it had no significant recovery effect for the widened trabecular separation (Tb.Sp) (**Figures 4B–F**). In parallel, the histological changes of the femur were examined using H&E staining. The results showed that the femoral tissues of OVX mice exhibited less trabecular bone, and a large number of lipid droplets were present instead. ZGBSF administration significantly reversed these alterations (**Figures 4G–I**). All these findings showed that ZGBSF effectively attenuated bone loss and the progression of OP.

### ZGBSF Promotes Osteogenesis and Fails to Inhibit the Formation of Osteoclasts

**Figure 5:**
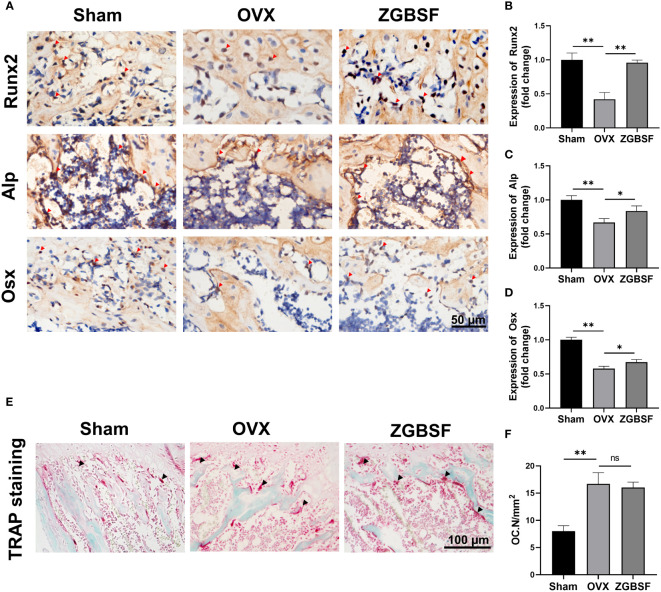
ZGBSF promotes osteogenesis and fails to inhibit osteoclastogenesis. **(A)** Immunohistochemistry of Runx2, Alp, and Osx of the distal femur. Red arrows indicated the high expression of Runx2, Alp, and Osx. **(B–D)** The quantification of Runx2, Alp, and Osx expression. **(E)** TRAP staining of the distal femur. **(F)** The number of osteoclasts (OC.N/mm^2^). The arrows represented the positive expression. Data were presented as mean ± SEM of three independent experiments. *P < 0.05, **P < 0.01, NS indicated no significant difference.

Bone homeostasis and remodelling is regulated by a balance between osteogenesis of osteoblast and osteoclastogenesis of osteoclast (37). To examine the effects of ZGBSF on osteogenesis, protein levels of Runx2, Alp, and Osx were determined by IHC analysis. The results of IHC analysis revealed that Runx2, Alp, and Osx were significantly decreased in OVX mice. The treatment of ZGBSF significantly increased the expression of Runx2, Alp, and Osx (**Figures 5A–D**). Subsequently, to explore the effect of ZGBSF on osteoclast formation, the number of osteoclasts was evaluated by TRAP staining. Compared with sham mice, the number of osteoclasts in the distal femurs of OVX mice was markedly increased. Unexpectedly, ZGBSF intervention had no significant effect on the increase of osteoclast number in OVX mice (**Figures 5E, F****)**.

### ZGBSF Suppresses Apoptosis and Activates the PI3K-Akt Signaling Pathway of Osteoblasts

**Figure 6:**
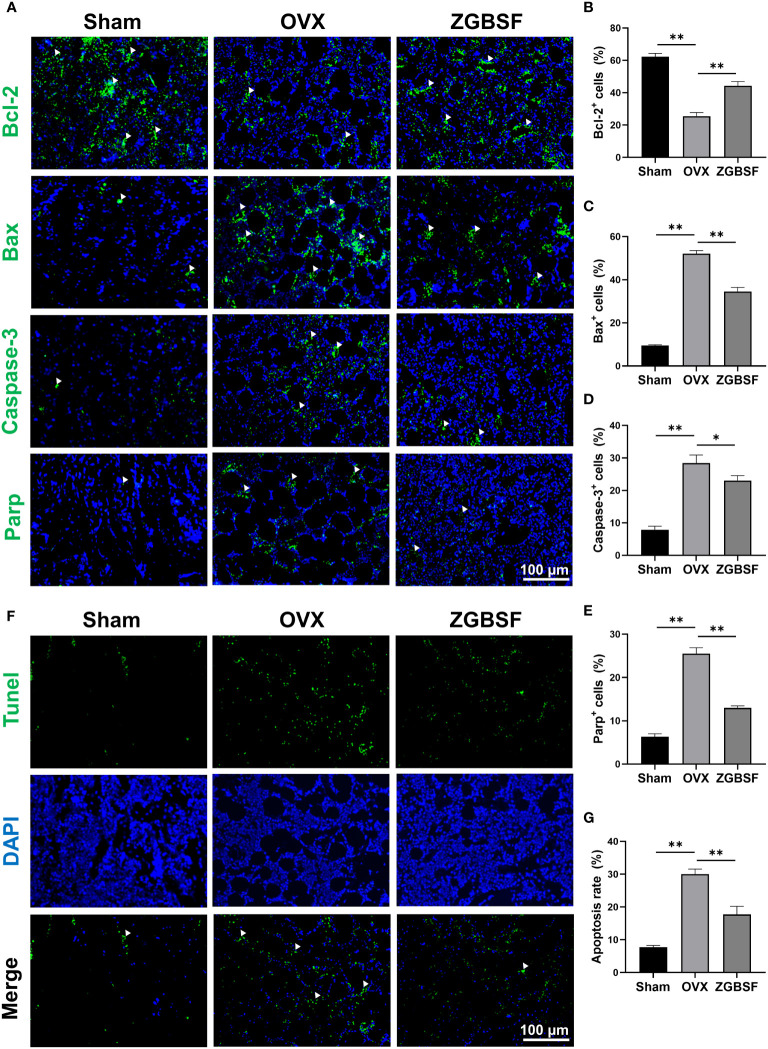
ZGBSF suppresses apoptosis of osteoblasts to ameliorate OP in OVX mice. **(A)** Immunofluorescence of Bcl-2, Bax, Caspase-3 and Parp in distal femur. **(B–E)** The quantification of Bcl-2, Bax, Caspase-3 and Parp positive cells. **(F)** Tunel staining was performed to examine the apoptosis status of osteoblasts. **(G)** The quantification of TUNEL positive rates in the distal femur. White arrows indicated the positive expression. Data were presented as mean ± SEM of three independent experiments. *P < 0.05, **P < 0.01.

The prediction results from our network pharmacology analysis revealed that the apoptosis and PI3K-Akt signaling pathways were pivotal biological events in ZGBSF against OP. Therefore, we first determined the expression of Bcl-2, Bax, Caspase-3, and Parp, indicators of apoptosis, by IF analysis. The IF results showed that Bcl-2 was markedly decreased in the distal femur of OVX mice, while Bax, Caspase-3 and Parp were significantly increased. ZGBSF significantly reversed these alterations (**Figures 6A–E**). In parallel, the anti-apoptosis effect of ZGBSF was further confirmed by TUNEL staining. The results of TUNEL staining revealed that ZGBSF significantly inhibited the increased apoptosis ratio of osteoblasts in femoral tissues of OVX mice (**Figures 6F, G****)**. All these findings showed that ZGBSF suppressed apoptosis of osteoblasts and contributed to osteogenesis, thus ameliorating bone loss of OVX mice.

**Figure 7:**
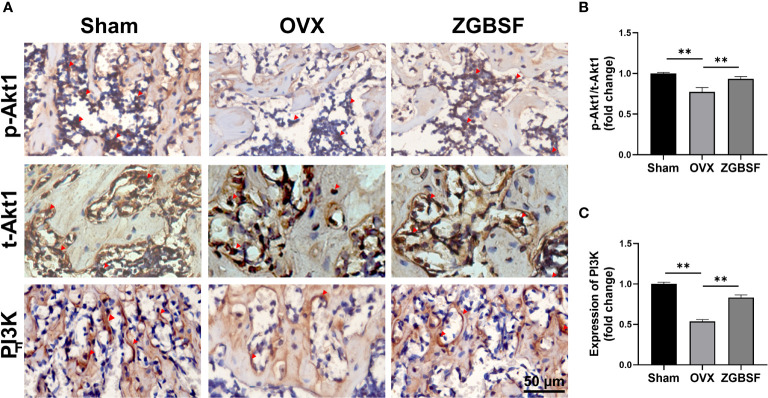
ZGBSF activates the PI3K-Akt signaling pathway of femoral tissue. **(A)** Immunohistochemistry of t-Akt1, Akt1(S473), and PI3K of distal femur. **(B, C)** The quantification of Akt1(S473)/t-Akt1, and PI3K expression. Red arrows indicated the positive expression. Data were presented as mean ± SEM of three independent experiments. **P < 0.01.

Next, to verify the effect of ZGBSF on PI3K−Akt signaling pathway predicted by KEGG enrichment analysis, the phosphorylation status of Akt1, and expression of PI3K protein were examined by IHC analysis of t-Akt1, p-Akt1, and PI3K. The IHC results showed that the levels of p-Akt1 and PI3K protein in distal femoral tissue of OVX mice were lower than those in sham mice. ZGBSF treatment significantly upregulated the expression of p-Akt1 and PI3K protein in ZGBSF mice (**Figures 7A–C**).

## Discussion

OP is a common chronic skeletal disease worldwide, characterized by bone loss and increased fracture risk. Fractures caused by OP are highly susceptible to disability and mortality in the elderly (38, 39). ZGBSF is a novel TCM formula, which has been proved to be an effective drug for treating OP in clinic. However, due to the diversity of components in ZGBSF, the pharmacological mechanism of its anti-OP effect is still elusive, which greatly hinders its popularization and application. Network pharmacology, a newly developed cross-discipline, has unique advantages in deciphering the molecular mechanisms of TCM on diseases with multiple components, targets, and pathways (40, 41). In this study, we deciphered the underlying molecular mechanisms of ZGBSF in the treatment of OP *via* network pharmacology analysis and *in vivo* experimental validation. We found that ZGBSF contributed to osteogenesis by inhibiting osteoblast apoptosis and activating PI3K-Akt signaling pathways, thereby attenuating bone loss and the progression of OP.

Based on network pharmacology analysis, the core compounds of TCM have made great contributions to the therapeutic effect of treating diseases (42). In the present study, we firstly discovered that the top active compounds in ZGBSF consist of quercetin, luteolin, kaempferol, baicalein, *etc*. Previous studies have shown that quercetin could inhibit oxidative stress and inflammatory response as well as the apoptosis of osteoblasts, thereby promoting osteogenesis (43). *In vivo* and *in vitro* experiments also showed that luteolin and kaempferol ameliorated OP by regulating oxidative stress and osteogenesis (44, 45). In addition, baicalein contributed to the expression of OP-related proteins and extracellular matrix mineralization (46). Therefore, all these findings showed that multiple components of ZGBSF had a good therapeutic effect on OP.

To explore the underlying mechanisms of ZGBSF in the treatment of OP, the OP-related targets of core components of ZGBSF were identified by the PPI network. We found that the top targets of ZGBSF for OP included Caspase-3, BCL2L1, and TP53, which were closely related to the process of apoptosis. Caspase-3 is a critical regulator of apoptosis, which has been proven to impair bone formation (47). A recent study reported that lowering the expression of Caspase-3 by quercetin and vitamin E inhibited osteoblast apoptosis, thus alleviating OP (48). BCL2L1 is a vital apoptosis regulator gene encoding splicing variants of anti-apoptosis [Bcl-x(L)] and pro-apoptosis [Bcl-x(S)] (49). Overexpression of BCL-x(L) in osteoblasts suppressed the apoptosis of osteoblasts and increased the bone mass of trabecular bone and cortical bone (50). In addition, as a pro-apoptotic protein, TP53 is closely related to OP (51). An *in vitro* experiment revealed that downregulating the expression of p53 observably increased the expression of osteogenesis-related proteins, such as Alp and Runx2 (52). All these findings showed that Caspase-3, BCL2L1, and P53 played an important role in the progression of OP.

Previous studies have shown that the excessive apoptosis of osteoblasts may exacerbate the progression of osteoporosis (53). Inhibiting the apoptosis of osteoblasts significantly enhanced osteogenic differentiation, thereby exerting anti-OP effects (54). A recent study on OP model of rats showed that quercetin improved ovariectomy-induced osteoporosis *via* regulation of apoptosis (48). Consistent with the above studies, our KEGG enrichment analysis showed that apoptosis was involved in the intervention of ZGBSF for OP, and *in vivo* mouse OVX model evidence revealed that ZGBSF treatment significantly overturned the decreased Bcl-2 protein and increased levels of Bax, Caspase-3, Parp as well as increased DNA damage in OVX mice. The results showed that ZGBSF attenuated the apoptosis of osteoblasts.

Increasing studies revealed that the PI3K-Akt signaling pathway played an important role in the physiological and pathological processes of OP (55, 56). Activation of the PI3K-Akt signaling pathway resulted in enhanced osteogenic differentiation of human bone marrow mesenchymal stem cells (15). Similarly, another work discovered that quercetin, one of the top active compounds in ZGBSF, promoted osteogenic differentiation and mineralization *via* PI3K-Akt signaling pathway (57). Consistent with those studies, our results uncovered that the activity of the PI3K-Akt signaling pathway was decreased in OVX mice, whereas ZGBSF treatment significantly activated the activity of the PI3K-Akt signaling pathway. And ZGBSF significantly enhanced the expression of decreased osteogenic markers (Runx2, Alp, and Osx) in OVX mice. However, ZGBSF administration had no significant effect on the increase of osteoclasts number in OVX mice. All these findings revealed that the treatment of ZGBSF dramatically promoted osteogenesis by activating the PI3K-Akt signaling pathway.

In total, ZGBSF could ameliorate the progression of OP *via* multi-ingredients, multi-targets, multi-pathways mode, which was different from a single drug with few targets. Network target and signaling pathway could perfectly decipher sophisticated interactions between diseases with chemical ingredients in TCM. However, there were some limitations in this study. First, only core targets and pathways of ZGBSF were verified, which may result in a slight deviation of results. Further validation of other relevant targets and signaling pathways predicted by network pharmacology is needed in future experiments. Second, our *in vivo* animal experiment was limited to mouse OVX model, so human-derived osteoblasts were used to make the experiment more clinically relevant in a follow-up experiment.

## Conclusion

In summary, to decipher the molecular mechanisms of ZGBSF in the treatment of OP, network pharmacology analysis coupled with experimental validation were performed. The network pharmacology analysis revealed that ZGBSF exerted anti-OP effects *via* multi-ingredients, multi-targets, and multi-pathways, and the experimental results validated that ZGBSF promoted osteogenesis *via* inhibition of osteoblast apoptosis and activation of PI3K-Akt signaling pathway, thus ameliorating bone loss of OP. This study could provide an optimized method to elucidate the pharmacological mechanisms of ZGBSF and supply a novel candidate for the treatment of OP.

## Data Availability Statement

The original contributions presented in the study are included in the article/supplementary material. Further inquiries can be directed to the corresponding authors.

## Ethics Statement

The animal study was reviewed and approved by The Ethics Committee of Zhejiang Chinese Medical University.

## Author Contributions

HR, PT, XL, CW contributed to the concept and design of this study. HZ, CZ, ZZ performed the computational analyses and performed the *in vivo* experiments. SaY, YB, FF, HL, YL, SxY, YG, YC, KZ, MY, WD, KT, HJ performed the experimental analysis. HZ drafted the manuscript. All the authors read, revised, and approved the final manuscript.

## Funding

This study was financially supported by National Natural Science Foundation of China (No.: 82174140, 82174401, 82104164, 81973870, 81973881, 81904053, 81804121), China Postdoctoral Science Foundation (No.: 2018M632154), Natural Science Foundation of Zhejiang Province (No.: LY22H270003, LBY22H270008, LY19H270006, and LQ19H080001), Traditional Chinese Medical Administration of Zhejiang Province (No.: 2022ZX005, 2022ZB119, 2021ZB090), Zhejiang medical and health science and technology project (No.: 2021KY222), Research Project of Zhejiang Chinese Medical University Scientific (No.: 2021JKZDZC02, 2021JKZKTS036A, 2021JKJNTZ022B, 2019ZG25, 2018ZR06), National Undergraduate Innovation and Entrepreneurship Training Program (202110344005, 202110344025, S202110344007, 202010344004), General Research Project of Zhejiang Provincial Education Department “Special Project for the Reform of Cultivation Mode of Professional Degree Graduate Students in Higher Education Institutions” (No.: Y202145932), Postgraduate Science Research Fund of Zhejiang Chinese Medical University (No.: 2021YKJ02, 2020YKJ07).

## Conflict of Interest

The authors declare that the research was conducted in the absence of any commercial or financial relationships that could be construed as a potential conflict of interest.

## Publisher’s Note

All claims expressed in this article are solely those of the authors and do not necessarily represent those of their affiliated organizations, or those of the publisher, the editors and the reviewers. Any product that may be evaluated in this article, or claim that may be made by its manufacturer, is not guaranteed or endorsed by the publisher.
